# Mixed‐methods approach to exploring patients’ perspectives on the acceptability of a urinary biomarker test in replacing cystoscopy for bladder cancer surveillance

**DOI:** 10.1111/bju.14690

**Published:** 2019-03-04

**Authors:** Wei Shen Tan, Chin Hai Teo, Delcos Chan, Malgorzata Heinrich, Andrew Feber, Rachael Sarpong, Jennifer Allan, Norman Williams, Chris Brew‐Graves, Chirk Jenn Ng, John D. Kelly, DETECT II trial collaborators, P Khetrapal, A ridhar, H Baker, F Ocampo, N Whotton, K Dent, S Pearson, J Hatton, M Newton, E Heeney, K Green, S Evans, M Rogers, A Dann, J Cook, M Cornwell, R Mills, H Knight, S Maher, A Rane, S Thomas, S Reyner, G Vallejera, P Adeniran, S Masood, S Ridgway, M Coulding, H Savill, J Mccormick, M Clark, G Collins, K Jewers, S Keith, G Bowen, J Hargreaves, K Riley, S Srirangam, R Mistry, J Chadwick, S Cocks, R Hull, A Loftus, L Dawson, H Roberts, C Main, S Jain, C Waymont, J Rogers, A Grant, V Carter, H Heap, C Lomas, P Cooke, Y Baird, S Moore, S Greenslade, J Margalef, I Chadbourn, M Harris, J Hicks, P Clitheroe, S Connolly, S Hodgkinson, H Haydock, A inclair, E Storr, L Cogley, S Natale, W Lovegrove, S Smith, K Smith, D Hewitt, R Sriram, K Atkinson, L Royle, J Madine, K MacLean, J Walsh, M Guerdette, M Hill, D Payne, A Power, J Cannon, L Devereaux, A Thompson, L Scarratt, T Hodgkiss, D Johnstone, J Johnson, J Allsop, J Rothwell, K Connolly, J Cherian, H Wardle, D Wilson, A Bayles, S Pelluri, J Pati, A Gherman, C Scott, S Madaan, A Taylor, E Edmunds, J Moore, A Rees, S Williams, S Caddy, S Dukes, A Goffe, K Buckhorn, L Nichols, P Acher, K Baillie, K Middleton, C Proctor, J Cresswell, A Chilvers, M Cain, A Vaux, D Watson, S Bradfield, H Gregory, H Mostafid, L Kehoe, S Drakeley, A Davies, L Williamson, R Krishnan, N Lunt, P Hill, H Burns, B Townley, L Wilkinson, H Wassall, J Hunt, B Holbrook, L Stancombe, J Morrison, L Vankoutrik, S Misra, G Fossey, A Richards, K Mcdonald, A Henderson, R Fennelly, M Tribbeck, K Ames, J Borwell, M Kotze, K Beesley, K Rennie, T Porter, A Gipson, L Piper, L Bailey, A Chrisopoulou, K Slevin, F McCartin, H Warburton, S Hathaway‐Lees, K Whetton, G Delves, A Day, T Bankole, S Broadhead, S Malde, M Oblak, D Ellis, S Bishara, T Barias‐Lara, I Donkov, H Thatcher, M Morris, C Culmsee, H Menzies, C Bartlett, C Cutting, N O'Brien, R Jannapureddy, A Kelkar, J Fitzgerald, S Longhurst, C Worth, M Peracha, S Mzazi, C Poile, L Griffiths, A Cook, N Barber, N Brar, A lty, B Zelhof, Rosie Blades

**Affiliations:** ^1^ Division of Surgery and Interventional Science University College London London UK; ^2^ Department of Urology University College London Hospital London UK; ^3^ Department of Primary Care Medicine Faculty of Medicine University of Malaya Kuala Lumpur Malaysia; ^4^ Health Behaviour Research Centre Department of Epidemiology and Public Health University College London London UK; ^5^ UCL Cancer Institute London UK; ^6^ Surgical and Interventional Trials Unit University College London London UK

**Keywords:** biomarker, diagnostic, patient‐reported outcome measure, questionnaires, semi‐structured interviews, #BladderCancer, #blcsm

## Abstract

**Objectives:**

To determine the minimal accepted sensitivity (MAS) of a urine biomarker that patients are willing to accept to replace cystoscopy and to assess qualitatively their views and reasons.

**Patients and Methods:**

Patients were part of a prospective multicentre observational study recruiting people with bladder cancer for a urine biomarker study (DETECT II; ClinicalTrials.gov: NCT02781428). A mixed‐methods approach comprising (1) a questionnaire to assess patients’ experience with cystoscopy and patients’ preference for cystoscopy vs urinary biomarker, and (2) semi‐structured interviews to understand patient views, choice and reasons for their preference.

**Results:**

A urine biomarker with an MAS of 90% would be accepted by 75.8% of patients. This was despite a high self‐reported prevalence of haematuria (51.0%), dysuria/lower urinary tract symptoms (69.1%) and urinary tract infection requiring antibiotics (25.8%). There was no association between MAS with patient demographics, adverse events experienced, cancer characteristics or distance of patients’ home to hospital. The qualitative analysis suggested that patients acknowledge that cystoscopy is invasive, embarrassing and associated with adverse events but are willing to tolerate the procedure because of its high sensitivity. Patients have confidence in cystoscopy and appreciate the visual diagnosis of cancer. Both low‐ and high‐risk patients would consider a biomarker with a reported sensitivity similar to that of cystoscopy.

**Conclusion:**

Patients value the high sensitivity of cystoscopy despite the reported discomfort and adverse events experienced after it. The sensitivity of a urinary biomarker must be close to cystoscopy to gain patients’ acceptance.

## Introduction

Bladder cancer is diagnosed in 8.0% of patients presenting with haematuria and, at initial diagnosis, 75% of cases are non‐muscle‐invasive bladder cancer (NMIBC) 1, 2. Patients with NMIBC have a 28–50% risk of recurrence and a 5–20% risk of progression at 5 years 3. The risk of recurrence necessitates regular surveillance cystoscopy, and guidelines recommend a risk‐adapted approach which can be as frequent as 3‐monthly cystoscopy, with lifelong follow‐up for high‐risk disease 4. The requirement for vigilant surveillance strategies is responsible for the high cost of healthcare associated with bladder cancer 5. Cystoscopy has a sensitivity of 98% for detection of bladder cancer although it remains an invasive procedure associated with patient discomfort and a 5% risk of UTI 6, 7.

The potential for urinary biomarkers for the detection of bladder cancer is an area of active research, and several biomarkers have been approved for use. Commercially available tests are licensed only as companion tests as they do not have the required diagnostic performance to replace cystoscopy (sensitivities of 57–82%) 8. Novel urinary biomarkers have reported high sensitivities but often lack robust prospective validation 9, 10. Regardless of the performance of current or future urinary‐based biomarkers, it is essential to understand patient perception and willingness to forgo cystoscopy for non‐invasive testing.

To understand patient views relating to cystoscopy and the potential to integrate urinary‐based biomarkers into a surveillance programme, we conducted a multicentre prospective observational study in which patients with NMIBC completed a questionnaire to determine the minimal accepted sensitivity (MAS) of a urine biomarker and we explored patients’ willingness to accept a biomarker rather than cystoscopy. We also report patient experience and adverse events during or after flexible cystoscopy. Reasons for preference were assessed through a qualitative analysis using semi‐structured interviews.

## Methods

### Study Design

Between September 2016 and April 2017, a total of 370 patients with histologically confirmed NMIBC and a minimum of 6 months’ (two surveillance cystoscopies and two urine collection for biomarker testing) follow‐up were recruited from 52 UK hospitals. Patients were sent questionnaires by post and 213 patients (57.6%) returned completed questionnaires. A total of 20 English‐speaking patients from this cohort consented to participate in a semi‐structured telephone interview. Of these patients, four (20%) had low‐grade cancers to ensure adequate representation of NMIBC. The full study protocol has been previously described 11. The reported study represents a secondary endpoint for the DETECT II study (clinicaltrials.gov: NCT02781428).

Patient demographics, education level attained, and history of bladder cancer were recorded. Cancer stage was assessed using TNM WHO cancer classification 12. Cancer risk was assessed using the European Association of Urology (EAU) risk classification 1. Distance from patients’ home to local hospital by private transport was calculated using www.maps.google.com.

### Patient Questionnaire

We constructed a patient experience questionnaire after consultation with the UCL Health Behaviour Research Centre (author M.H.) as no validated questionnaire exists. The questionnaire domains assessed overall experience of cystoscopy, anxiety preceding cystoscopy and pain experienced, using a five‐point Likert‐scale. Preference for cystoscopy or urinary biomarker was assessed using the standard gamble method 13. Cystoscopy was defined as having a sensitivity of 98% 6. The MAS for a urinary biomarker was defined as the sensitivity at which patients expressed either a preference for a urine biomarker or were neutral about accepting either the biomarker or cystoscopy.

### Qualitative Semi‐Structured Interview

Each qualitative semi‐structured interview lasted between 20 and 40 min. All interviews were carried out by one interviewer and patients were approached after a minimum of 6 months’ follow‐up from initial cancer diagnosis. The interview was designed to explore the following: experience of cystoscopy; perceived advantages and disadvantages of cystoscopy; perceived advantages and disadvantages of a urine biomarker; reasons for the preference for cystoscopy or a urine biomarker; perceived acceptable sensitivity of a urine biomarker for detection of bladder cancer; and preference for a surveillance pathway combining a urine biomarker interspaced with cystoscopy.

### Statistical Methods and Data Analysis

Continuous data were reported using descriptive statistics such as mean, median, interquartile range and 95% CI. Categorical variables were compared using the chi‐squared test. The *t*‐test and anova were used to compare the mean of continuous variables. spss v22 (IBM Corp, Armonk, New York, NY, USA) was used for statistical analysis and statistical significance was set at *P* < 0.05.

Patient interviews were performed using Skype (Microsoft, Redmond, WA, USA) and recorded using an Evaer Skype recorder. Interview recordings were transcribed, and data were managed using Nvivo 11 (QSR International, Melbourne, Vic., Australia). A thematic analysis was used, open coding was performed by two researchers (W.S.T. and C.H.T.) on the first two transcripts and differences were resolved by discussion. Codes were assigned to sentences/paragraphs of transcripts based on the study objective. Axial coding was performed, and existing codes combined to create larger themes. One researcher (W.S.T.) continued to code remaining transcripts and any new emerging codes were discussed. Comparisons were made throughout the analysis to form the final framework. Background notes throughout all study phases were reviewed to avoid potential bias in reporting.

### Ethics and Informed Consent

The corresponding author certifies that, when applicable, a statement(s) has been included in the manuscript documenting institutional review board, ethics committee or ethical review board study approval; principles of Helsinki Declaration were followed in lieu of formal ethics committee approval; institutional animal care and use committee approval; all human subjects provided written informed consent was obtained from all patients. DETECT II study protocol received Health Research Authority: London‐Stanmore Research Ethics Committee approval on 30 August 2016 (IRAS project ID: 203022, REC reference: 16/LO/1044). This trial is registered on clinicaltrials.gov: NCT02781428.

## Results

### Patient Demographics

A patient cohort flow diagram is shown in Fig. S1. Baseline characteristics and clinical‐pathological variables for 213 patients are shown in Table 1. The median patient age was 74.0 years and 167 patients (78.4%) were men. Patients with a primary diagnosis of NMIBC in the preceding 6 months accounted for 62.9% of the cohort (*n* = 134). A total of 74.1% of patients (*n* = 158) had ≤5 previous cystoscopies. High‐risk NMIBC according to EAU risk classification was confirmed in 40.3% of patients (*n* = 83), but only 17.8% of patients (*n* = 38) perceived their cancer as high risk.

**Table 1:** Patient demographics and clinical‐pathological variables (*N* = 213)

Variable	
Age, median (IQR), years	74.0 (67.1–81.1)
Men, *n* (%)	170 (79.8)
Highest education level, *n* (%)	
No formal education	8 (3.8)
High school	56 (26.3)
GCSE	39 (18.3)
A‐level	20 (9.4)
University or higher degree	31 (14.6)
Not known	59 (27.7)
Smoking history, *n* (%)	
Non‐smoker	56 (26.3)
Ex‐smoker	129 (60.6)
Current smoker	18 (8.5)
Not known	10 (4.7)
Ethnicity, *n* (%)	
White	188 (88.3)
Non‐white	6 (2.8)
Not known	19 (8.9)
Employment, *n* (%)	
Full time/part‐time/home maker/voluntary	45 (21.1)
Retired	161 (75.6)
Disability/unemployed	4 (1.9)
Missing	3 (1.4)
New or recurrent tumour, *n* (%)	
New	135 (63.4)
Recurrence	78 (36.6)
Procedure, *n* (%)	
TURBT/bladder biopsy	206 (96.7)
Cystodiathermy	7 (3.3)
Previous cystoscopies, *n* (%)	
≤2	66 (31.0)
2–5	92 (43.2)
≥6	47 (22.1)
Not known	8 (3.8)
Tumour grade, *n* (%)	
G1	36 (16.9)
G2	99 (46.5)
G3	71 (33.3)
Not known	7 (3.3)
Tumour stage, *n* (%)	
CIS	3 (1.4)
pTa	156 (73.2)
pT1	47 (22.1)
Not known	7 (3.3)
Papillary with concurrent CIS, *n* (%)	5 (2.4)
Disease risk, *n* (%)	
Low	18 (8.5)
Intermediate	105 (49.3)
High	83 (39.0)
Not known	7 (3.3)
Patients perception of disease risk, *n* (%)	
Low	49 (23.0)
Intermediate	112 (52.6)
High	38 (17.8)
Not known	14 (6.6)

CIS, carcinoma *in situ*.

### Patient‐Reported Adverse Events

**Figure 1:**
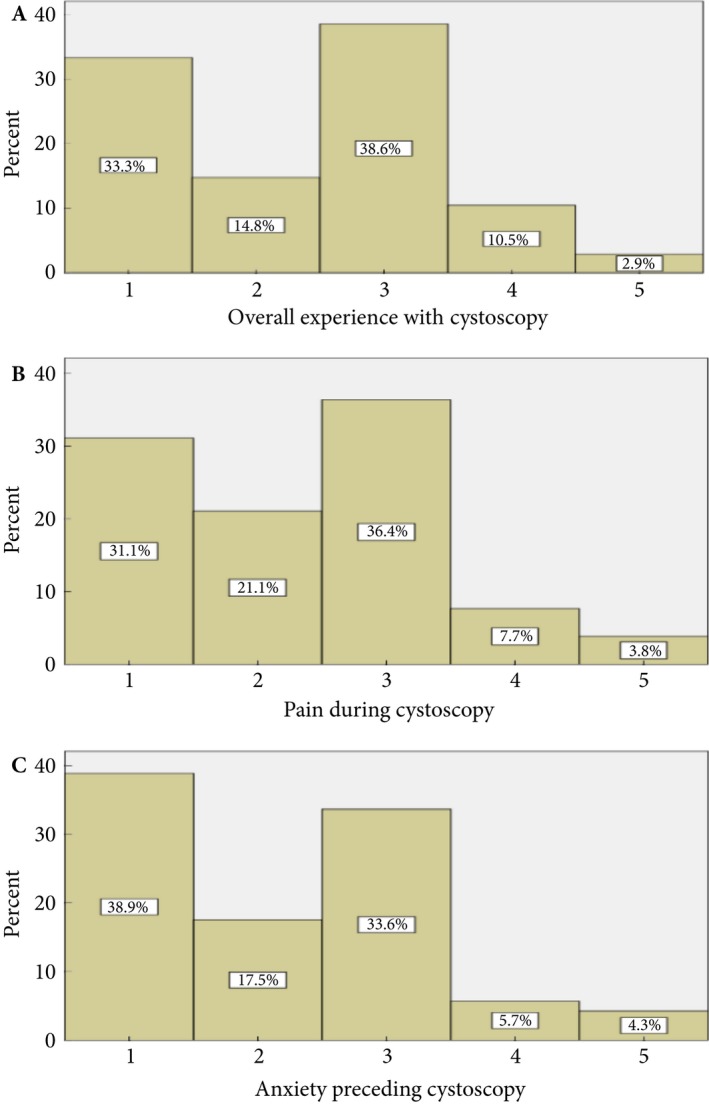
Patient experience after cystoscopy: (**A**) overall experience, (**B**) pain during cystoscopy, (**C**) anxiety preceding cystoscopy. 1 denotes no symptoms/painless/not anxious. 5 denotes severe symptoms/very painful/very anxious.

The majority of patients experienced an adverse event after cystoscopy, with 77.5% (*n* = 165) reporting ≥1 adverse event (Table 2). The self‐reported prevalence of haematuria, dysuria/LUTS and UTI requiring antibiotics after cystoscopy was 46.9% (*n* = 100), 67.1% (*n* = 143) and 23.1% (*n* = 51), respectively. The significance of symptoms after cystoscopy is highlighted by the fact that 13.4% of patients had an unpleasant experience after the procedure. Moderate to significant pain was reported by 11.5% of patients and 10.0% of patients reported moderate to significant anxiety preceding cystoscopy (Fig. 1).

**Table 2:** Complications experienced after cystoscopy (*n* = 213)

Adverse event	*n* (%)
Any adverse event	
Yes	165 (77.5)
No	47 (22.1)
Not known	1 (0.4)
Haematuria	
Yes	100 (46.9)
No	96 (45.1)
Not known	17 (8.0)
Dysuria/urinary symptoms	
Yes	143 (67.1)
No	64 (30.0)
Not known	6 (2.8)
UTI requiring antibiotics	
Yes	51 (23.9)
No	147 (69.1)
Not known	15 (7.0)

### Minimum Acceptable Sensitivity for a Urine Biomarker to Replace Cystoscopy

A total of 78.6% (*n* = 163) of patients would be happy to use a urine biomarker if it was as sensitive as cystoscopy (Table 3), with 24.2% of patients prepared to accept a urine biomarker even if sensitivity was as low as 85%. There was no relation between patient experience of cystoscopy and MAS. Most patients (74.2%, *n* = 158) would accept the option to interspace cystoscopy with a urine biomarker to extend the time interval between surveillance cystoscopies.

**Table 3:** Minimal acceptable sensitivity for acceptance of urinary biomarker

MAS	*n* (%)
85%	50 (23.5)
90–95%	26 (12.2)
96–97%	30 (14.1)
98%	57 (26.8)
Preference for cystoscopy regardless of accuracy of cystoscopy	44 (20.7)
Missing	6 (2.8)

MAS, minimal acceptable sensitivity.

There was no difference in MAS according to patient demographics, previous adverse events or experience during or after cystoscopy, cancer characteristics or distance to hospital from home (Table 4). There was a trend towards significance between a lower MAS for men (*P* = 0.052) and patients without a recurrence in the previous 6 months (*P* = 0.078; Table 4). Patient confidence in the accuracy of cystoscopy was the top reason for choosing cystoscopy (70.5%), followed by reassurance by a clinician (51.9%) and a preference for a diagnostic test performed in hospital (51.4%; Table S1). The top reasons for choosing a urine biomarker was previous discomfort after cystoscopy (30.5%), avoiding a hospital visit (28.6%) and the non‐invasive nature of the test (28.1%).

**Table 4:** Patient demographics and clinical‐pathological variables stratified according to minimal acceptable sensitivity

Variables	Minimal acceptable sensitivity					*P*
	85%	90–95%	96–67%	98%	Cystoscopy regardless	
Median (IQR) age, years	73.4 (67.9–81.9)	71.6 (66.0–78.1)	72.7 (64.7–80.7)	75.5 (65.5–79.8)	76.9 (69.5–83.1)	0.486
Men	47 (94.0)	19 (73.1)	21 (70.0)	45 (78.9)	33 (75.0)	0.052
Highest education level						
No formal education	0 (0)	1 (3.8)	1 (3.3)	4 (7.0)	2 (4.5)	0.165
High school	14 (28.0)	6 (23.1)	11 (36.7)	13 (22.8)	12 (27.1)	
GCSE	6 (12.0)	4 (15.4)	5 (16.7)	16 (28.1)	7 (15.9)	
A‐level	4 (8.0)	5 (19.2)	3 (10.0)	4 (7.0)	2 (4.5)	
University or higher degree	8 (16.0)	7 26.9)	2 (6.7)	9 (15.8)	4 (9.1)	
Not known	18 (36.0)	3 (11.5)	8 (26.7)	11 (19.3)	17 (38.6)	
Previous cystoscopies, *n* (%)						
≤2	15 (30.0)	7 (26.9)	16 (53.3)	14 (24.6)	12 (27.3)	0.114
2–5	17 (34.0)	16 (61.5)	8 (26.7)	30 (52.6)	20 (45.5)	
≥6	15 (30.0)	3 (11.5)	6 (20.0)	11 (19.3)	10 (22.7)	
Not known	3 (6.0)	0 (0)	0 (0)	2 (3.5)	2 (4.5)	
New or recurrent cancer						
New	28 (58.0)	19 (73.1)	21 (70.0)	32 (56.1)	30 (68.2)	0.411
Recurrent	21 (42.0)	7 (26.9)	9 (30.0)	25 (43.9)	14 (31.8)	
Previous recurrence within 6 months						
Yes	30 (60.0)	20 (76.9)	17 (56.7)	39 (68.4)	28 (63.6)	0.078
No	10 (20.0)	4 (15.4)	11 (36.7)	13 (22.8)	15 (34.1)	
Not known	10 (20.0)	2 (7.7)	2 (6.7)	5 (8.8)	1 (2.3)	
Tumour grade						
G1	9 (18.0)	8 (30.8)	5 (16.7)	9 (15.8)	5 (11.4)	0.231
G2	24 (48.0)	7 (26.9)	18 (60.0)	29 (50.9)	17 (38.6)	
G3	15 (30.0)	11 (42.3)	6 (20.0)	16 (28.1)	21 (47.7)	
Not known	2 (4.0)	0 (0)	1 (3.3)	3 (5.3)	1 (2.3)	
Tumour stage						
CIS	2 (4.0)	0 (0)	0 (0)	1 (1.8)	0 (0)	0.428
pTa	40 (80.0)	17 (65.4)	21 (70.0)	43 (75.4)	30 (68.2)	
pT1	6 (12.0)	9 (34.6)	8 (26.7)	10 (17.5)	13 (29.5)	
Not known	2 (4.0)	0 (0)	1 (3.3)	3 (5.3)	1 (2.3)	
Actual disease risk						
Low	5 (10.0)	4 (15.4)	2 (6.7)	5 (8.8)	2 (4.5)	0.482
Intermediate	26 (52.0)	9 (34.9)	18 (60.0)	31 (54.4)	18 (40.9)	
High	17 (34.0)	13 (50.0)	9 (30.0)	18 (31.6)	23 (52.3)	
Not known	2 (4.0)	0 (0)	1 (3.3)	3 (5.3)	1 (2.3)	
Patient's presumed disease risk						
Low	16 (32.0)	3 (11.5)	5 (16.7)	9 (15.8)	14 (31.8)	0.353
Intermediate	22 (44.0)	14 (53.8)	18 (60.0)	34 (59.6)	24 (54.5)	
High	9 (18.0)	8 (30.8)	5 (16.7)	12 (21.1)	4 (9.1)	
Not known	3 (6.0)	1 (3.8)	2 (6.7)	2 (3.5)	2 (4.5)	
Any adverse event						
Yes	41 (82.0)	17 (65.4)	23 (76.7)	45 (78.9)	36 (81.8)	0.504
No	9 (18.0)	9 (34.6)	7 (23.3)	12 (21.1)	8 (18.2)	
Haematuria						
Yes	28 (56.0)	11 (42.3)	12 (40.0)	24 (42.1)	22 (50.0)	0.484
No	20 (40.0)	14 (53.8)	14 (46.7)	30 (52.6)	17 (38.6)	
Not known	2 (4.0)	1 (3.8)	4 (13.3)	3 (5.3)	6 (11.4)	
Dysuria/LUTS						
Yes	33 (66.0)	17 (65.4)	19 (63.3)	39 (68.4)	32 (72.7)	0.764
No	17 (34.0)	9 (34.6)	10 (33.3)	17 (29.8)	10 (22.7)	
Not known	0 (0)	0 (0)	1 (3.3)	1 (1.8)	2 (4.5)	
UTI requiring antibiotics						
Yes	9 (18.0)	4 (15.4)	10 (33.3)	13 (22.8)	13 (29.5)	0.196
No	39 (78.0)	19 (73.1)	19 (63.3)	42 (73.7)	25 (56.8)	
Not known	2 (4.0)	3 (11.5)	1 (3.3)	2 (3.5)	6 (13.6)	
Overall experience						
No symptoms	23 (46.0)	11 (42.3)	12 (41.4)	31 (54.4)	22 (51.2)	0.833
Neutral	20 (40.0)	9 (34.6)	13 (44.8)	19 (33.3)	17 (39.5)	
Severe symptoms	7 (14.0)	6 (23.1)	4 (13.8)	7 (12.3)	4 (9.3)	
Pain						
Painless	24 (48.0)	13 (50.0)	15 (50.0)	27 (47.4)	27 (61.4)	0.82
Somewhat painless	19 (38.0)	9 (34.6)	11 (36.7)	22 (38.6)	15 (34.1)	
Very painful	5 (10.0)	4 (15.4)	4 (13.3)	7 (12.3)	2 (4.5)	
Not known	2 (4.0)	0 (0)	0 (0)	1 (1.8)	0 (0)	
Anxiety preceding cystoscopy						
Not anxious	27 (54.0)	14 (53.8)	17 (56.7)	32 (56.1)	26 (59.1)	0.972
Somewhat anxious	17 (34.0)	8 (30.8)	10 (33.3)	19 (33.3)	15 (34.1)	
Very anxious	5 (10.0)	4 (15.4)	3 (10.0)	6 (10.5)	3 (6.8)	
Not known	1 (2.0)	0 (0)	0 (0)	0 (0)	0 (0)	
Median (IQR) distance from clinic, miles	6.3 (3.4–11.2)	9.9 (3.8–13.2)	6.1 (3.7–12.6)	5.5 (3.5–11.8)	6.4 (3.3–11.5)	0.726

CIS, carcinoma *in situ*.

### Qualitative Analysis

The demographics and tumour characteristics of the 20 patients interviewed are shown in Table S2. Main themes that emerged are shown in Table 5.

**Table 5:** Qualitative analysis for advantages and disadvantages of cystoscopy, advantages of urine test, active comparison between cystoscopy and urine test and patient skepticism about urine test

**Advantages of cystoscopy**
**Visual diagnosis**
‘It's [bladder cancer] caught on camera as it were. You can literally see what's going on’
‘I know it's there and it's [bladder cancer] staring right at me. You literally see and discuss what's there to the chap or women who is doing it’
‘The fact that the camera shows you that thing on the camera and that they show a scar and they do a grand tour of my bladder. It's reassuring to see that’
**Confidence in diagnostic accuracy**
‘I think it's quite accurate. I would say 95%. It found mine and mine was really tiny, a couple of mm. They picked it up with the camera and there it was sitting on the wall. It looked like a little sea anemone’
‘They actually have a camera on the end and its magnified they can see anywhere in the bladder. So, to me, that's accurate. If it wasn't, they wouldn't have found mine’
‘I presume it's because it's on the screen. I mean I'm not a doctor. I can only assume that what you see is what you get sort of thing. I mean, there it is…. it's a cancer. And there is this on the screen I suppose that must be 100% identifiable’
‘To be honest, I think it [cystoscopy] is the only way we can know for sure is there anything there or not’
**Tolerability of cystoscopy**
‘Cystoscopy is something I got used to. I have had quite a few of those now and I accept that fully’
‘Well I don't like them, but I want to have the most accurate diagnosis possible’
‘If it's the only way then it's best to know what's going on but it's not exactly a great overall experience. So, if there's an alternative obviously, it's preferable’
‘It is quite invasive, but I think I preferred that because I think then you know that you got an accurate reading of what's going on’
‘It's embarrassing obviously because the thought of exposing yourself to people but it's necessary at the same time. So it's overcoming one thing or the other’
‘Well I don't suppose there is any other way to do it’
‘You know I really have no dignity left for the start. But you know it's a small price to pay’
‘I mean even though a bit of uncomfortable, I don't mind having the camera probe’
**Instant diagnosis**
‘I can literally walk out of there knowing that all is well and that's very helpful’
‘..that's [cystoscopy] quite a reassurance to walk out of there thinking that, that month was all right and we go on from here’
‘…. it was a bit daunting, but it was instantaneous. There wasn't any waiting around’
‘I can see all in front of me. I guess when someone does a blood test you've got to wait 2–3 weeks for the results to come back’
**Qualified person**
‘But if you have a qualified person who takes a look inside your bladder with a camera. That's as good as any I think’
‘They do it at the very professional way and it's a reassurance for me’
‘…the person who did it said it was all clear, so you know… nothing else to go off really’
**Disadvantages of cystoscopy**
**Invasive**
‘Well the thought of a camera going inside me from the place they put it in my urethra….the thought of that going into me does put me off. I don't really like that, but you know where else can it go…. I feel that the best entry point…. not being cut you open’
‘It was just, obviously the fact that my tube had been invaded with an alien piece… It was just that I have never gone through anything like this before and it was not what I was expecting…’
**Adverse events**
‘The urine was burning at first but that goes away after a couple of hours… So, what I do is I drink plenty of water and just flush it all through’
‘….I did have a bladder problem where I couldn't control the use of my bladder whereas when I had a feeling there…. I had to pee you know straight away… Rushing to the toilet is most cases’
‘It was the after effects that went wrong. I couldn't pass no water but that's sorted itself out now’
‘Well it must have been sort of the first couple of passes, a little bit of blood came out after that, it was okay’
**Embarrassing**
‘It's embarrassing obviously because the thought expose yourself to people but it's necessarily at the same time. So it's overcoming one thing or the other’
‘Again it's a psychological embarrassing feeling that well okay I'm exposing myself to somebody.. ’
‘You know I really have no dignity left for the start’
**Operator dependent**
‘…the doctor was being shown how to use a new machine. Two other people watching and a lady showing her what to do you know. It didn't bother me at the time but you know I was a bit sore. I was well quite sore after that. It took me a week or so to get myself right with it. Now the second, the next time I went for it, it was the registrar… I couldn't believe it. He did it and he said right okay thank you. So, I said, “are we done” and he said, “yeah”. With the other lady, it seemed like an hour, but this was probably about 10 min. Well, it was only minutes with this chap. I just walked out as good as when I went in’
**Advantages of urine test**
**No adverse events**
‘If they can find it all the way through urine test… that would be a much better and more comfortable way of checking’
‘..certainly more convenient and less uncomfortable for the patient’
**Less intrusive**
‘…if it helps detect cancer in a less invasive way I suppose it is good’
‘.. there's less interference in there,… I've always been a bit sore when I've had one [cystoscopy]. You know you go for a wee and it sore’
‘… the urine test is much less alarming thing to do than going in for a cystoscopy’
‘… reduce the level of personal invasions’
**Quicker treatment time**
‘…where you could start treatment early I think would be a great advantage’
**Reduce patient embarrassment**
‘It's not a very nice experience you know…there were two young girls in their 20s. Nurses. It's not a nice experience anyway but having that it's a bit…. you know, not nice’
**Convenience**
‘I suppose logically the urine test if it's proven is a bit easier’
‘… it would be a simple thing to collect some urine and see you could determine whether there were cancer cells, where you could start treatment early I think would be a great advantage’
‘Obviously a lot easier than the cystoscopy’
‘…reduce inconveniencing the patient’
‘…if I could find out everything from urine sample then it would be a lot easier because you don't have to spend any time in the hospital’
‘..certainly more convenient and less uncomfortable for the patient’
**Active comparison between cystoscopy and urine biomarker**
‘Given the particular cancer I have is high grade…signet cell variation…. I'd be wary of it [urine biomarker]. I need more reassurance as I am not out of the woods yet Because I'm just a year into a disease. Everything is happening well for me at present the treatment seems to be working well for me and I'm very relaxed and confident about it. I would need some reassurance that this is as good or comparable’
‘Well I am not fussed either way…that [urine biomarker] would be an easier way obviously instead of going through cystoscopy but I don't know how accurate it is going to be…If you do pull it off then all well and good’
‘I'll look at it differently. You know I'm 79, a realist… I am content, happy with the treatment. And the cystoscopy is something that has become part of my life and I'm content with that’
‘Well I still would like it [urine biomarker] to be up to 99 percent. It has to be. You can't mess with peoples’ life. You can't have 70–78% and then it's quite possible you missed it. You know. if its 99–99.5 percent, at least you are in the right area with cystoscopy’
‘I think it must be comparable to the cystoscopy. Otherwise, the numbers that could slip through would be unfortunate’
‘Well there is no 100% guarantee here, but a high percentage would be good’
‘Even if the percentages weren't as good, I would prefer to have the urine test. It's [cystoscopy] not a very nice experience you know. The last time I went, there's two young girls in their 20s. Nurses. It's not a nice experience anyway but having that is not nice’
‘Like I said, I don't really believe in…Well I mean if you can positively detect cancer in that fashion then it will be so good. But I like I like physical checks as well so like I said that the cystoscopy often a good idea too’
‘Cystoscopy, you could only see a visual… the urine test could detect earlier then the visual one…. you could only see as far as the eye can see’
‘I don't know. I am not a doctor. I would follow the advice of the doctor, wouldn't you? ’
‘I'm happy with this is cystoscopy because I know it's working’
‘I would always prefer whichever is most accurate’
‘I want as much of certainty as possible’
‘I would just like to know definitely rather than not so sure’
**Patient skepticism about urine test**
‘I would need some reassurance that this is as good or comparable’
‘You will need to reassure me with the evidence. I suppose logically the urine test if it's proven which is a bit easier. But I'm not moved yet to trust it. Not without some evidence’
‘Yeah the percentages don't weigh up at the moment, really. You know, if it's only a 40 percent chance of success, I would stick to the other I would? So I was more uncomfortable but if we put up with it you will be sure it will be alright’
‘I worried that the water sample, whether that would be as good as the cystoscopy’
‘I'm sure it [use of urine biomarker] will happen. Maybe not yet. I'm happy with this is cystoscopy because I know it's working’
‘What is the aim? I imagine it is to cut down the number of flexible cystoscopy which is very expensive in terms of hospital time and staff time and so forth. Is that the basis of it? ’
‘I think it [urine biomarker] will still be playing at the back of my mind whether it was accurate or not’
‘Also, the severity… I had a very mild one, a very small growth. As someone with a more aggressive and bigger…then it might be better with the to have a camera’

### Views and Experience of Cystoscopy

Patients appreciate the fact that cystoscopy provides a visual diagnosis of cancer and attributed this to a presumed near‐perfect sensitivity for the detection of bladder cancer. While patients did not like cystoscopy, they were prepared to tolerate it because of its good diagnostic ability. Patients also valued the fact that cystoscopy provides an instant diagnosis and appreciated that a healthcare professional performs the cystoscopy.

Patient perception of passing a cystoscope along the urethra was that it can be disturbing, although they recognize the requirement to visualize the bladder. Some patients described the procedure as embarrassing and felt violated after cystoscopy. Patients also appreciate that cystoscopy performed by an experienced urologist would reduce adverse events and patient discomfort.

### Views and Experience of Urine Testing

Patients valued the convenience of a urine biomarker, reducing the need to attend hospital and be subjected to a procedure. Furthermore, patients appreciated that a urine biomarker was free of adverse events, unlike cystoscopy, and believed that a urine biomarker would allow earlier testing and subsequently allow prompt commencement of treatment.

### Active Comparison Between Cystoscopy and Urine Test

When comparing between cystoscopy and the urine biomarker, patients were pragmatic and understood that no test is 100% accurate. Patients prioritized the test with the highest sensitivity and most would only accept a urine test with a similar sensitivity to cystoscopy. Missing bladder cancer during surveillance was a significant worry to patients, and patients with high‐grade bladder cancer felt particularly concerned about missing recurrence and prioritized the high sensitivity of cystoscopy. Some patients’ familiarity with cystoscopy and the fact that they had a positive experience with cystoscopic detection of cancer reinforced their preference for cystoscopy over a urine test. An overarching theme was that patients were not confident in the ability of a urine test to identify bladder cancer with a high sensitivity as they perceived it to be ‘experimental’ when compared to cystoscopy, the current ‘gold standard’.

Patients who had previously experienced embarrassment related to cystoscopy were willing to accept a lower diagnostic sensitivity for a biomarker. All patients were open to interspacing cystoscopy with a urine biomarker to increase the interval between cystoscopies, although most reinforced the requirement for comparable sensitivity. Some patients expressed the opinion that a molecular urine test may potentially identify cancers before they are diagnosed visually. Further, some patients were skeptical about the ability of a biomarker which would be able to match cystoscopy.

## Discussion

This represents the first study to explore qualitatively the views and decision‐making of patients when considering between cystoscopy and a urine biomarker for the detection of bladder cancer recurrence. The majority of patients (75.8%) recognized biomarker performance as important and would discount any biomarker with MAS <90%; however, 63.3% of patients would accept a biomarker with a MAS of ≥95%, suggesting that patients’ willingness to accept a biomarker is linked to its performance characteristics. Nevertheless, 21.3% of patients would prefer cystoscopy regardless of urine biomarker performance because of factors such as immediate readout and clinician interaction despite having to travel to a hospital.

Patient acceptance of cystoscopy was independent of experience of adverse events relating to the test, suggesting that the high sensitivity of cystoscopy is of paramount importance. This is similar to data from colonoscopy, where patients consider a high sensitivity to be of paramount importance with risk of adverse effects a secondary concern 14. Our data suggest that the prevalence of complications after cystoscopy are not negligible, with 50% of patients’ self‐reporting haematuria and urinary symptoms after the procedure and 24% developing UTI requiring antibiotics. This is considerably higher than previous reports, although it represents a cumulative experience of patients who have had multiple cystoscopies and not an incidence rate 7.

We did not observe an association between patient demographics, education level and clinical‐pathological variables with a lower MAS. In addition, higher disease stage, grade, actual risk or patient‐perceived risk classification was not associated with a higher acceptable sensitivity. The data indicate that even patients with a lower risk of recurrence or progression place a high emphasis on accurate cancer detection. Patients have a perceived benefit for early detection of recurrence which is clear in high‐risk bladder cancer, although limited data exist to support this in low‐risk cancers. Observational reports suggest that active surveillance of patients with G1 pTa NMIBC does not increase oncological risk 15. Distance to hospital was not a factor, as patients value the visit to hospital and prefer seeing a clinician as it reassures them.

Two previous studies have reported the MAS required of a urine biomarker to replace cystoscopy in patients with NMIBC in the surveillance setting. Vriesema et al. 16 surveyed 102 patients with at least 12 months’ follow‐up and reported that 89% of patients would not accept a urinary biomarker with a sensitivity of <90%. Yossepowitch et al. 17 assessed the preference of 200 patients undergoing check cystoscopies at various time points and reported that 75% of patients would not accept a biomarker with an accuracy of <95%. Both studies reported that men were willing to accept a marginally lower MAS 16, 17. The study by Vriesema et al. and Yossepowitch et al. 16, 17 report that patients who were older (>67 years) and those who experienced a higher pain intensity after cystoscopy, respectively, were significantly more likely to accept a lower sensitivity.

The present study differs from these two studies. Besides reporting the MAS of a urine biomarker that patients were prepared to accept, we also investigated the reasons for patients’ preference for cystoscopy or urine biomarker using qualitative and quantitative methods. We did not find any variables associated with a lower MAS. A reason for this may be the fact that patients in the present study completed the questionnaire 6 months following a cancer diagnosis, suggesting a shorter time interval compared with the other two studies (minimum of 12 months 16 and median follow‐up of 50 months 17). The fear of cancer recurrence may be prioritized over pain attributed to cystoscopy 16, 17. Patients in both studies also did not experience the use of a urine biomarker and assumed that cystoscopy was 100% sensitive, which it is not in clinical practice.

Patients valued the visual element of cystoscopy as this was something they could relate to, and the absence of a visual diagnosis of cancer provides significant reassurance. Patients are aware that cystoscopy is the gold standard diagnostic test for bladder cancer surveillance and perceived a urine test as experimental, which may have introduced bias; however, if the sensitivity of a urine test were proven to be close to cystoscopy, 78.7% of patients would be happy to accept a urine test at intervals.

Limitations of the present study should be acknowledged. Complications such as UTI requiring antibiotics after cystoscopy were self‐reported by patients and not confirmed by urine culture. In addition, the present study was not designed to report the incidence rate of UTI per cystoscopy performed, but to identify correlation between patient‐reported and perceived adverse events and acceptability of a urine biomarker test. Further, all patients had at least one general anaesthetic for cystoscopy or transurethral resection of bladder cancer and, while the questionnaire was intended to gauge patient's perspectives on surveillance flexible cystoscopy, the patient's overall experience with any cystoscopy may have had an influence in shaping their opinion. It was assumed that patients would comprehend the questionnaire when completing it, as no medical terminology was used. Although all patients provided a urine sample for biomarker testing, they were not provided with the results of the biomarker. The knowledge of the biomarker results combined with a patients’ experience with cystoscopy may affect the MAS. Finally, cystoscopies were nearly universally performed by urology trainees/residents and in more experienced hands, adverse events and patient's acceptability may be improved.

In conclusion, we report that patients with NMIBC valued the high sensitivity that cystoscopy accords despite the reported patient discomfort and adverse events experienced. Patients considered any diagnosis of cancer to be significant and were not willing to compromise on the diagnostic ability of a test. Hence, a diagnostic sensitivity of any urine biomarker must be close to that of cystoscopy before patients are prepared to accept it over cystoscopy.

## Conflict of Interest

John D. Kelly and Andrew Feber are involved in the development and validation of a urine biomarker ‘UroMark’ which is the primary objective of the current DETECT II study.

**Abbreviations**
EAU: European Association of Urology
MAS: minimal accepted sensitivity
NMIBC: non‐muscle‐invasive bladder cancer

## Supporting information


**Appendix S1**. DETECT II patient perspectives questionnaire.
**Appendix S2**. Patient Interview outline.
**Table S1.** Reasons for selecting cystoscopy or urinary test.
**Table S2.** Patient demographics and tumor characteristics of patients interviewed.
**Figure S1.** Flow chart.

## Appendix 1 DETECT II Collaborators

Participating centres and investigators (*principle investigators at each centre): WS Tan, P Khetrapal, A Sridhar, H Baker, F Ocampo, JD Kelly* (UCLH), N Whotton, K Dent, S Pearson, J Hatton, M Newton, E Heeney, K Green, S Evans, M Rogers* (Northern Lincolnshire & Goole NHS Foundation Trust), A Dann, J Cook, M Cornwell, R Mills* (Norfolk & Norwich University Hospital), H Knight, S Maher, A Rane* (East Surrey Hospital), S Thomas, S Reyner, G Vallejera, P Adeniran, S Masood* (Medway Maritime Hospital), S Ridgway, M Coulding, H Savill, J Mccormick, M Clark, G Collins* (Tameside General Hospital), K Jewers, S Keith, G Bowen, J Hargreaves, K Riley, S Srirangam* (East Lancashire Hospitals NHS Trust), R Mistry, J Chadwick, S Cocks, R Hull, A Loftus, L Dawson*, (Royal Bolton Hospital), H Roberts, C Main, S Jain* (St James's University Hospital), C Waymont, J Rogers, A Grant, V Carter, H Heap, C Lomas, P Cooke* (New Cross Hospital), Y Baird, S Moore, S Greenslade, J Margalef, I Chadbourn, M Harris, J Hicks* (Western Sussex Hospitals NHS Foundation Trust), P Clitheroe, S Connolly, S Hodgkinson, H Haydock, A Sinclair* (Stepping Hill Hospital), E Storr, L Cogley, S Natale* (Derriford Hospital), W Lovegrove, S Smith, K Smith* (King's Mill Hospital), D Hewitt, R Sriram* (University Hospitals Coventry), K Atkinson, L Royle, J Madine, K MacLean* (Royal Cornwall Hospital), J Walsh, A M Guerdette, M Hill, D Payne* (Kettering General Hospital), A Power, J Cannon, L Devereaux, A Thompson* (Royal Albert Edward Infirmary), L Scarratt, T Hodgkiss, D Johnstone, J Johnson, J Allsop, J Rothwell, K Connolly, J Cherian* (The Pennine Acute Hospitals NHS Trust), H Wardle, D Wilson, A Bayles* (University Hospital of North Tees), S Pelluri, J Pati* (Homerton Hospital), A Gherman, C Scott, S Madaan* (Darent Valley Hospital), J A Taylor, E Edmunds, J Moore* (East Sussex Healthcare NHS Trust), A Rees, S Williams, S Caddy, S Dukes, A Goffe* (Dorset County Hospital), K Buckhorn, L Nichols, P Acher* (Southend Hospital), K Baillie, K Middleton, C Proctor, J Cresswell, A Chilvers, M Cain, A Vaux, D Watson* (James Cook University Hospital), S Bradfield, H Gregory, H Mostafid* (Royal Surrey County Hospital), L Kehoe, S Drakeley, J A Davies, L Williamson, R Krishnan* (Kent & Canterbury Hospital), N Lunt, P Hill, H Burns, B Townley, L Wilkinson, H Wassall, A Sinclair* (Macclesfield Hospital), J Hunt, B Holbrook, L Stancombe, J Morrison, L Vankoutrik, S Misra* (North Devon District Hospital), G Fossey, A Richards, K Mcdonald, A Henderson* (Maidstone Hospital), R Fennelly, M Tribbeck, K Ames, J Borwell* (Salisbury District Hospital), M Kotze, K Beesley, K Rennie, T Porter* (Yeovil District Hospital), A Gipson, L Piper, L Bailey, A Chrisopoulou, K Slevin, F McCartin, H Warburton* (Wythenshawe Hospital), S Hathaway‐Lees, K Whetton, G Delves* (Queen's Hospital), A Day, T Bankole, S Broadhead, S Malde* (Guy's Hospital), M Oblak, D Ellis, S Bishara, T. Barias‐Lara, I Donkov* (West Middlesex University Hospital), H Thatcher, H M Morris, C Culmsee, A H Menzies, C Bartlett, C Cutting* (Royal Lancaster Infirmary), N O'Brien, R Jannapureddy, A Kelkar* (Barking, Havering and Redbridge University Hospitals NHS Trust), J Fitzgerald, S Longhurst, C Worth, A M Peracha* (Royal Derby Hospital), S Mzazi, C Poile, L Griffiths* (Leicester Royal Infirmary), A Cook, N Barber, N Brar, A Alty, B Zelhof, Rosie Blades* (Royal Preston Hospital).
