# Supplementary material for: Mixed‐methods approach to exploring patients’ perspectives on the acceptability of a urinary biomarker test in replacing cystoscopy for bladder cancer surveillance
Source: BJU Int. 2019 Mar 4;124(3):408–17. doi: 10.1111/bju.14690 (PMC6767410; doi:10.1111/bju.14690)
Supplement: Supplementary file 1 — Appendix S1. DETECT II patient perspectives questionnaire. Appendix S2. Patient Interview outline. Table S1. Reasons for selecting cystoscopy or urinary test. Table S2. Patient demographics and tumor characteristics of patients interviewed. Figure S1. Flow chart. [file BJU-124-408-s001.doc]

### Supplementary files not for publication

### Appendix 1: DETECT II patient perspectives questionnaire

### Appendix 2: Patient Interview outline

You have been invited to take part in this study as part of the DETECT II study. As part of this study, you have been having a cystoscopy which is the standard investigation to monitor your bladder and detect any recurrence of cancer. You are also providing a urine sample for a new test which may also be able to detect recurrence of cancer.

The aim of this interview is to explore your experience of being diagnosed with bladder cancer and having a cystoscopy and the urinary test as methods of monitoring for cancer recurrence.

I would like to begin by asking questions about your condition

**Accessing knowledge of bladder cancer**

1) What is your understanding about your cancer? (causes, treatment effectiveness, ways to manage it etc.)

2) How long do you think will your condition last?

**Wellbeing**

3) How does your illness affect your wellbeing? (well-being, activities of daily living, social roles, work, the use of healthcare services, experiencing symptoms or side effects of treatment)

**Assessing experience of cystoscopy for bladder cancer**

4) What do you think is the best way to monitor your cancer

5) What do you think about cystoscopy as a method of bladder cancer monitoring?

6) How did you find the experience of having to have cystoscopy?

7) How frequently do you think you will be having cystoscopies from now on?

8) How does it make you feel?

9) How accurate to you think cystoscopy is in terms of detecting cancer?

**Attending cystoscopy appointments**

10) How do you think you will find attending these appointments at the proposed time-intervals?

**Assessing experience using the urine collection kit**

11) what do you think about the urine test?

12) What do you think about the urine test you had to do as part of the trial?

13) How did you find providing the urine sample and mailing it back (easy or difficult)?

**Access confidence in using urine test for bladder cancer**

14) How does the urine test compare with having a cystoscopy?

15) How good would a urine test need to be before you would be happy to accept it instead of cystoscopy?

16) How would you feel about the test as being a standard monitoring method for detecting cancer recurrence instead of cystoscopy?

If NO, please explain why. How accurate do you think the urinary test would need to be in detecting cancer before you would accept it?

17) Consider abbreviated standard gamble

If the urinary test detects will miss (X of 100 bladder cancers (vs misses 2 of every 100 cancers in cystoscopy), would you prefer the urinary test or cystoscopy?

18) If the urine test had similar accuracy to cystoscopy in terms of ability to spot bladder cancer, would you agree to replace all your cystoscopies with the urine test?

**Assessing opinion of urine a urine test to reduce the frequency of cystoscopy**

19) What do you think about urine the urine test to increase the interval between cystoscopies. Ie:

If YES - how often would you like to have urinary test between your cystoscopies? why at these particular intervals?

If NO, please explain why? How accurate do you think the urinary test would need to be in detecting cancer before you would accept it?

20) Consider abbreviated standard gamble

21) What is your view of using both urine test and cystoscopy to check for bladder cancer recurrence?

22) If it was up to you how often would you like to have a cystoscopy?
